# Effect of 1-ethyl-3-(3-dimethylaminopropyl) carbodiimide and *N*-hydroxysuccinimide concentrations on the mechanical and biological characteristics of cross-linked collagen fibres for tendon repair

**DOI:** 10.1093/rb/rbv005

**Published:** 2015-05-16

**Authors:** Zafar Ahmad, Jennifer H. Shepherd, David V. Shepherd, Siddhartha Ghose, Simon J. Kew, Ruth E. Cameron, Serena M. Best, Roger A. Brooks, John Wardale, Neil Rushton

**Affiliations:** ^1^Orthopaedic Research Unit, Department of Surgery University of Cambridge, Addenbrooke’s Hospital, Cambridge, CB2 0QQ, UK; ^2^Department of Materials Science and Metallurgy, University of Cambridge, Cambridge, CB3 0FS, UK; ^3^Tigenix Ltd, Cambridge, CB4 0FY, UK

**Keywords:** biopolymer, scaffolds, soft tissue

## Abstract

Reconstituted type I collagen fibres have received considerable interest as tendon implant materials due to their chemical and structural similarity to the native tissue. Fibres produced through a semi-continuous extrusion process were cross-linked with different concentrations of the zero-length cross-linker 1-ethyl-3-(3-dimethylaminopropyl) carbodiimide (EDC) in combination with *N*-hydroxysuccinimide (NHS). Tensile properties of the fibres were considered, along with imaging of both surface structure and fibrillar alignment. Resistance of the fibres to bacterial collagenase was investigated and fibre sections seeded with human tendon cells for biological characterization, including cell adhesion and proliferation. The work clearly demonstrated that whilst the concentration of EDC and NHS had no significant effect on the mechanics, a higher concentration was associated with higher collagenase resistance, but also provided a less attractive surface for cell adhesion and proliferation. A lower cross-linking concentration offered a more biocompatible material without reduction in mechanics and with a potentially more optimal degradability.

## Introduction

Tendons can be considered as unidirectional fibre-reinforced composites and serve to transmit the forces generated by muscle contraction with a minimal dispersion of energy [1]. Whilst the principal role of tendon must be to resist tension, a certain degree of compliance is also necessary [1]. These conflicting demands are resolved as a result of the hierarchical structure of tendon, the ‘crimped’ nature of the collagen fibres [1] and the contrasting nature of the stiff collagen and the viscous, highly hydrated and proteoglycan-rich surrounding matrix [2]. In addition to their mechanical demands, tendons must also support cellular activity [3, 4].

Unfortunately damage to tendon is both prevalent and debilitating and the limited blood and nerve supply results in a slower healing rate than many other tissues [5–7]. It has been shown that scar tissue formation is significant and this has both a distorted composition and structure compared with uninjured tissue and as such inferior biomechanics [8, 9]. Surgery for tendinopathy or rupture is considered a last resort and typically consists of simple repair techniques such as sutures and soft tissue anchors[6]. Allograft material provides an alternative, but concerns include the risk of disease transmission, bacterial infection and immunogenic response as well as escalating demand [5]. Decellularized biological extra-cellular matrix material and non-degradable polymer fibre systems have also been considered for tendon and ligament repair with mixed results, mechanics often proving a limiting factor [10–17].

Reconstituted type I collagen fibres have attracted considerable interest as tendon implant materials, as a result of their chemical and structural similarity to the native tissue [18]. In two studies, reconstituted collagen tendon prostheses were developed and implanted in the rabbit Achilles tendon [19, 20]. The prosthetics appeared highly successful, showed significant degradation, rapid formation of neotendon and exhibited mechanical properties approaching those of native tendon [19, 20].

In 1989, Kato and Silver first described the extrusion of an acidic suspension of insoluble collagen under aqueous conditions on a laboratory scale [21]. Since then, considerable effort has been focused on optimization of the mechanical and biological characteristics of these fibres, [22–26]. The acidic collagen suspension is typically extruded into a bath containing neutral fibre forming buffer (FFB); the collagen gels on contact with neutral pH as the fibrils reconstitute and the macroscopic fibres form. Along the length of the bath the thread dehydrates due to the osmotic gradient between the collagen and the FFB [27].

Whilst early studies considered the reconstitution of collagen fibres from suspensions of insoluble collagen, it was later suggested that these fibres may not fully replicate the hierarchical substructure of native connective tissue [28]. Early investigations of *in vitro* collagen fibrillogenesis [29, 30] had suggested that soluble type I collagen could be self-assembled into fibrous collagen scaffolds with both mechanical properties and fibrillar sub-structure comparable to native tissue. There thus became an emphasis on the investigation of collagen extrusion from solutions of soluble collagen [3, 28, 31–34]. It is these fibres that have been analysed to the greatest degree, particularly in terms of fibrillar structure; however, an interest in constructs produced from insoluble collagen has remained [23, 24, 26, 35, 36] largely as a result of the significantly lower cost of the insoluble material.

When collagen is generated *in vivo*, cross-linking occurs both enzymatically and non-enzymatically and covalent inter and intra-molecular bonds are formed [37]. It is these cross-links that are essential for mechanical strength and proteolytic resistance and these cross-links are not formed during the self-assembly or reconstitution of collagen in neutral pH. Cross-linking routes include chemical (glutaraldehyde, isocyanates or carbodiimide based) [31], physical (dehydrothermal treatment) [38, 39] and enzymatic [40]. The zero length cross-linker carbodiimide 1-ethyl-3-(3-dimethylaminopropyl) carbodiimide (EDC) in combination with *N*-hydroxysuccinimide (NHS) has been shown to be an effective route for the cross-linking of extruded collagen fibres [22, 24, 26, 41]. This route results in a lower cross-linking density than other chemical and physical cross-linking routes but it has been shown to exhibit a favourable biological response [34] unlike some more aggressive techniques that have been associated with inflammatory response and reduced bioactivity [42].

A recent study considered the implantation of cross-linked collagen fibres into the central-third of the patellar tendon in an ovine model [24]. The carbodiimide-cross-linked implants showed increased tissue ingrowth compared with the other cross-linking chemistry considered, but after 6 months implantation, very little of the implant was observed to be replaced by regenerated tissue. This resulted in an implant region with a lower mechanical strength than an ‘empty’ central defect after the 6 month period. It was suggested that the level of cross-linking was too high and that an optimal balance between mechanical properties and degradation was essential. Although *in vitro* studies have shown EDC-cross-linked fibres to demonstrate favourable behaviour in terms of oriented fibroblast migration compared with other cross-linking routes, they none-the-less showed a decrease compared with non-cross-linked material [34].

Previous work by the authors [41] has shown cross-linking of collagen fibres using the EDC/NHS chemistry to result in a significant increase in tensile mechanics compared with the uncross-linked material. Another study by the authors used atomic force microscopy (AFM) to demonstrate clear fibrillar structure in the case of a heavily cross-linked sample, whilst the same structure did not appear evident in a fibre without cross-linking [36]. However, neither study investigated the effect of cross-linking concentration. Clearly, value could be gained by an investigation into the effect of concentration of the carbodiimide cross-linking agent on the degradation, mechanical and biological characteristics of collagen fibre constructs designed for tendon repair.

This study therefore considers the carbodiimide cross-linking of collagen fibre constructs produced by a semi-continuous extrusion process from an insoluble collagen suspension. The combination of EDC and NHS cross-linking is considered at a number of different concentrations with fibre mechanics, fibrillar structure and resistance to collagenase investigated. Fibre sections were also seeded with human tenocytes for biological characterization, including cell adhesion and proliferation.

## Materials and Methods

### Collagen fibre extrusion

**Figure 1:**
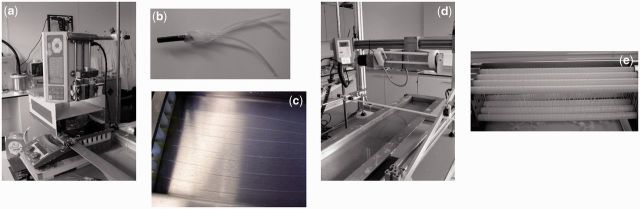
Extrusion of collagen fibres: collagen slurry drawn into two syringes and using a vertically oriented syringe pump (**a**), the slurry extruded through a three channel manifold system (**b**), to form six channels flowing along the length of the FFB bath (**c**). The six strands are then collected together at the end of the bath and wound onto a spool (**d**). Fibre bundles are allowed to dry overnight on the spools (**e**) before cross-linking and/or washing.

Collagen fibre bundles were extruded using a method applied previously by the authors [22, 24, 41], summarized in the photographs of Fig. 1 and based upon the route of Silver *et al.* [43]. Briefly, a 6 mg/ml collagen slurry in 2 mM HCL was produced from acid-swollen gel collagen derived from bovine dermis (Devro Medical, Moodiesburn, Scotland) and drawn into two syringes, each with a three-thread manifold attached (Fig. 1b). The collagen was then extruded at a controlled rate into a flowing bath of FFB, forming six separate strands (Fig. 1c). The FFB consisted of a 20% wt/v solution of polyethylene glycol (molecular weight 8000) in phosphate-buffered saline (PBS) solution. This PEG concentration has been previously applied by the authors [22, 23, 36, 41] and was shown by Zeugolis *et al.* [33] to be the optimal amount required for reproducible fibres. Once the six strands reached the end of the FFB bath, they were collected together and wound onto a rotating spool in the form of a six ply fibre. Horizontal motion of the spool meant a continuous length of fibre was produced, largely without overlap. 15 ml of collagen slurry was used per fibre construct, resulting in a collagen mass of ∼90 mg. The collagen fibre construct was left on the spool to dry overnight.

### Cross-linking

Cross-linking agents were the water soluble *N*-(3-Dimethylaminopropyl)-*N*′-ethylcarbodiimide hydrochloride (EDC) and NHS both purchased from Sigma Aldrich. Previously, the authors have used 25 mM EDC and 12.5 mM NHS in an 80/20 acetone/PBS solution mixture for cross-linking of these fibre constructs [41]. The vessel used for cross-linking was such that 3 litres of cross-linking solution were used per 10 fibre bundles and cross-linking time was 2 h. It has been shown that this corresponds to an excess of the cross-linking agents compared with the number of carboxylic acid groups present ([41] with reference to [44]) and as a result lower cross-linking concentrations were considered in this current work. Three cross-linking concentrations referred to as: 25 mM (25 mM EDC and 12.5 mM NHS), 2.5 mM (2.5 mM EDC and 1.25 mM NHS) and 0.25 mM (0.25 mM EDC and 0.125 mM NHS) were used as well as fibres without any cross-linking (referred to as 0 mM). In all instances, 3 litres of cross-linking solution were used per 10 fibre constructs and the cross-linking time maintained at 2 h. After cross-linking, the constructs underwent a multi-stage washing process with two, 30 min washes in PBS solution and two, 30 min washes in high purity deionized water. Fibre constructs where cross-linking was not applied, underwent the multi-stage washing process to ensure full removal of the polyethylene glycol. All collagen constructs were air dried overnight, before being manually detached from the spool.

### Characterization

#### Mechanical testing

Quasi-static tensile loading was carried out on individual sections of fibre, after hydration in PBS for at least 1 h. At least three measurements of diameter were made per fibre using a digital micrometer, with the mean value taken and a circular cross-section assumed. Mean fibre diameters varied between 50 and 400 µm (mean 132 µm, standard deviation 95 µm) with no significant variation with different cross-linking concentrations (results not shown). An Instron 3343 Universal Testing machine (Instron, High Wycombe, UK) was used with fibre-specific grips and the initial gauge length again measured using a micrometer. Initial gauge length was ∼10 mm in all cases so as to avoid any length effects. At least five fibres were tested for each cross-linking condition. Engineering stress—strain curves were generated and the ultimate tensile strength, strain to failure and tangential modulus at 5% strain calculated.

#### Imaging of collagen fibre constructs

Small sections of fibre were imaged using scanning electron microscopy (SEM) or AFM. Samples for SEM were secured to an aluminium stub with carbon tape, sputter-coated with a thin layer of gold and viewed under a JEOL 820 SEM in secondary electron mode. For AFM, fibre sections were placed onto 10 × 5 mm microscope slides using silver dag, and imaging was carried out using the Bruker Dimension 3100 AFM in tapping mode with an MPP-11100-10 probe. Multiple regions of 2 × 2 µm were considered. Images of fibres cross-linked with 0.25 mM cross-linking concentration were compared with those previously taken of standard cross-linked fibres and those without any cross-linking (imaged using multimode AFM with Nanoscope III control). Only fibres with this low cross-linking concentration were imaged so as to investigate whether fibrillar structure was maintained even with significant reduction in concentration.

#### Collagenase experiment

An evaluation of the resistance of the fibres to degradation with bacterial collagenase was carried out based upon the method of Weadock *et al.* [45]. Three fibres from each group (25, 2.5, 0.25 and 0 mM) were added to 25 ml, 0.2% bacterial collagenase solution (> 125 Collagen digestion units (CDU), from Sigma Aldrich) (filtered sterilized) made in complete DMEM (Dulbecco’s Modified Eagle Medium, supplemented with 10% foetal calf serum, 1 µg/ml amphotericin, 10 µg/ml gentamycin, 100 IU/ml penicillin, 100 µg/ml streptomycin and 2 mM L-glutamine). These were checked initially hourly for the first day, and thereafter daily for 21 days.

#### Tendon cell preparation

Discarded semi-tendinosus tendons of humans from anterior cruciate ligament (ACL) reconstructions were taken with permission (Cambridgeshire 2 Research Ethics Committee—ref: 06/Q0108/213) and washed in antibiotic medium (DMEM supplemented with 10% foetal calf serum, 1000 IU/ml penicillin, 1 mg/ml streptomycin, 10 µg/ml amphotericin and 100 µg/ml gentamycin), for 1 h. The tendon was then minced as finely as possible using a scalpel and added to 25 ml 0.2% collagenase solution, made in complete DMEM. This was incubated for 12 h on a shaker, before the cell suspension was removed and centrifuged. The pellet was washed in fresh medium to remove any residual collagenase and re-suspended in a culture flask. Cells were allowed to adhere and then the medium changed every 2-3 days. Confluency of the cells was normally achieved after ∼2 weeks. Cells were trypsinized and passaged on a weekly basis, with cells at passage three normally applied for cell culture.

#### Cell culture on collagen fibres

Uniform sections of the fibre construct (8 mm lengths) were hydrated with PBS and placed into the wells of a 96 well plate. Each sample was seeded with 10^5^ cells in 50 µl DMEM taking care to pipette onto the material. The same number of cells were also seeded in micro-masses on 96 well plates in triplicate for controls. After seeding, samples were incubated for 5 h, before 150 µl of cell medium was added to each well. For each condition, seeding was carried out in triplicate. Cells were cultivated in standard conditions (37°C, humidified, 5% CO_2_), with the medium changed every third day. Samples were re-plated at every medium change with cells and culture plates harvested by snap freezing at 7, 14 and 21 days.

##### Cell number

Scaffolds were trypsinized and cells resuspended in fresh medium. This medium was then diluted with PBS and the number of cells measured using a Millipore Sceptre Cell Counter.

##### Cell proliferation assay

Cultured scaffolds were incubated overnight in papain digest buffer (0.1 M phosphate buffer pH 7, 10 mM L-cysteine, 2 mM EDTA, 125 µg/ml Papain) in order to extract DNA from the cells on the scaffold. Extracts were measured for total DNA content using the Hoechst assay [46].

##### SEM of cultured scaffolds

The fibre sections (with or without cells, after being incubated for 1 week in medium) were washed and fixed in 4% glutaraldehyde in 0.1 M PIPES buffer at pH 7.4. They were then post-fixed in 1% osmium ferricyanide, dehydrated in increasing ethanol concentrations, critical-point dried and finally mounted onto aluminium stubs and sputter coated with gold. Imaging was carried out using an FEI-Philips XL30 FEG SEM (Philips, UK).

### Statistics

Data were analysed for statistical significance between the different cross-linking concentrations with a single factor Analysis of variance (ANOVA) followed by Tukey’s post-hoc testing (SPSS software, IBM). Statistical significance was established at *P* ≤ 0.05.

## Results

### Physical characterization

**Figure 2:**
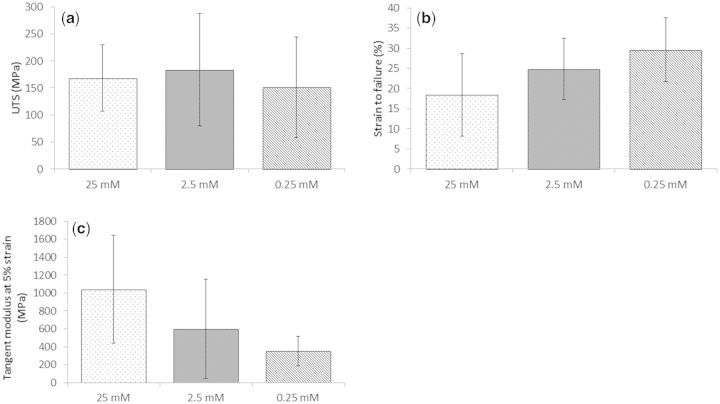
Effect of cross-linking on single fibre mechanics: (**a**) ultimate tensile strength, (**b**) strain to failure, (**c**) tensile modulus defined at 3-5% strain (*n* = 5-7; mean ± SD). No statistically significant difference was observed with cross-linking concentration on any of the three parameters considered.

Tensile loading demonstrated that the concentration of the EDC and NHS could be reduced by 100 times from the standard cross-linking concentration without influence on the ultimate tensile strength of the fibres. Whilst the data of Fig. 2 did not demonstrate statistically significant variation, it is at least indicative of a slight reduction in modulus and increase in strain to failure as a result of the reduced cross-linking concentration. Variation within the data was large, perhaps a result of fibre inhomogeneity due to the continuous fibre extrusion process [47] and the relatively limited number of samples (*n* = 5-7). In all cross-linking conditions a significant deviation in fibre diameters were observed, but no difference in mean diameter was observed between groups and mechanics were independent of fibre diameter (results not shown). Whilst the author’s previous study clearly showed EDC/NHS cross-linking to significantly increase mechanical properties of fibre constructs [41], this current study has shown that less extreme cross-linking conditions can be considered without detriment to mechanics.

**Figure 3:**
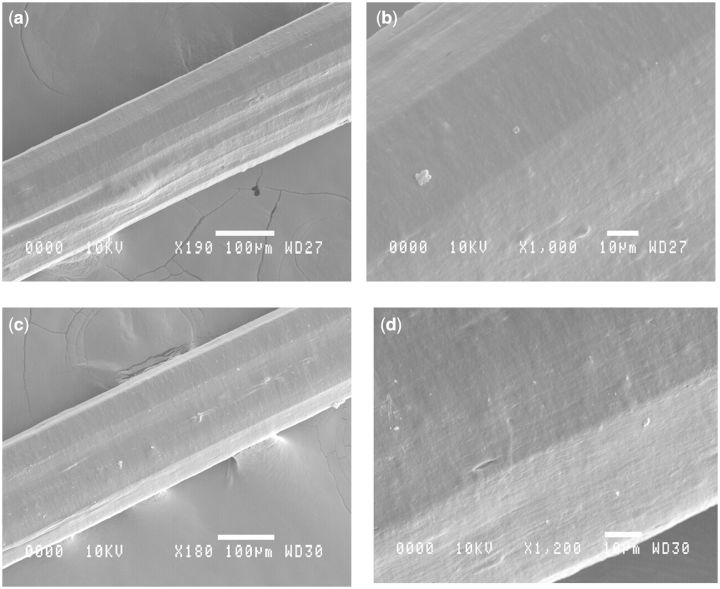
SEM images of sections of extruded collagen fibre: (**a, b**) without any cross-linking and (**c, d**) with the standard 25 mM EDC and 12.5 mM NHS cross-linking.

**Figure 4:**
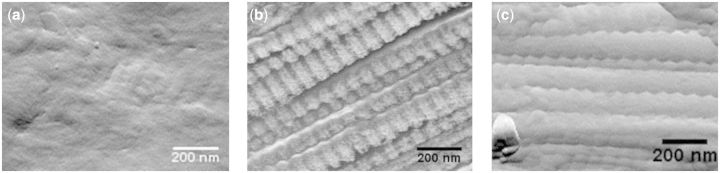
AFM images of collagen fibre sections: (**a**) without cross-linking, (**b**) 1/100 cross-linking concentration, (**c**) standard cross-linking concentration.

Fibre appearance or structure appeared unaffected by the degree of cross-linking. As demonstrated in the SEM images of Fig. 3 even a comparison of no cross-linking and the most aggressive standard cross-linking showed no appreciable difference in surface morphology or gross fibre structure. AFM demonstrated that even fibres cross-linked with the lowest EDC/NHS cross-linker concentrations exhibited the characteristic banding pattern of collagen fibrils as observed in native tissue (Fig. 4b and c). The degree of alignment and order was high regardless of the concentration of the cross-linking agent. The degree of order was much less evident when fibres were not cross-linked and as previously reported [36] the characteristic banding was seen only in very localized regions (Fig. 4a). The AFM imaging in this study in combination with the author’s previous work [36] demonstrates that collagen fibres extruded from insoluble collagen demonstrate the same fibrillar sub-structure as observed during the fibrillogenesis [48] of soluble tendon and indeed observed in native type I collagen.

### Collagenase experiment

All three non-cross-linked fibres (0 mM group) had entirely degraded 6 h after being put into the collagenase. By the 48 h time point the 2.5 mM and 0.25 mM cross-linked fibres had also degraded, however, the fibres cross-linked with the standard cross-linking chemistry (25 mM) were still present 21 days after being placed in the collagenase.

### Biological analysis

**Figure 5:**
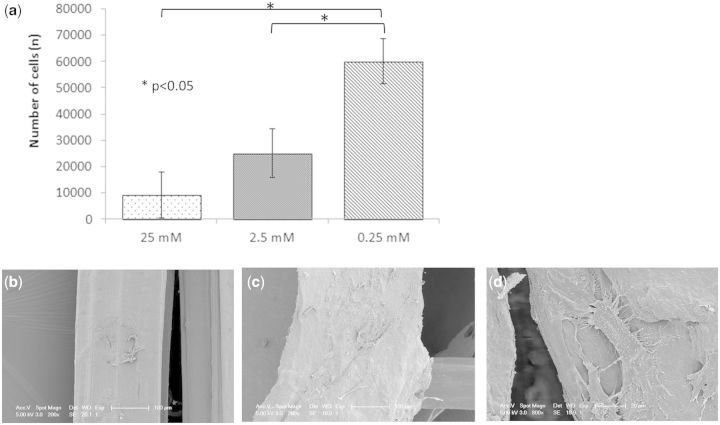
(**a**) number of human cells adherent to fibres with the different cross-linking concentrations after 24 h (*n* = 3; mean ± SD); (**b, c**) SEM images of tenocytes adherent to fibres with standard cross-linking and 1/100 cross-linking, respectively; (**d**) higher magnification image of tenocytes on fibre with the lowest cross-linking demonstrating the flattened morphology and cellular processes.

**Figure 6:**
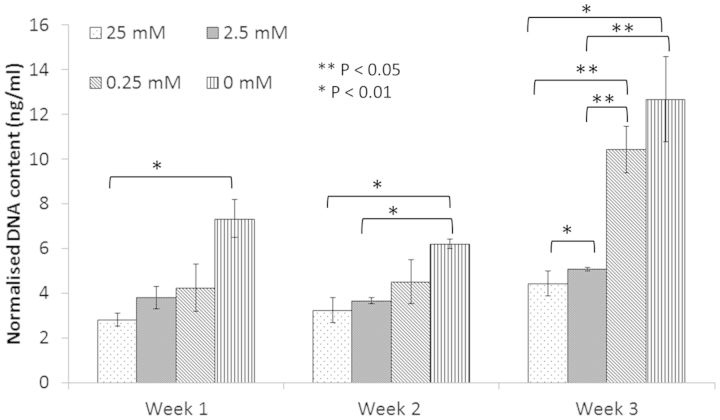
Amount of DNA extracted from human tenocytes seeded on fibres of the various cross-linking concentrations (*n* = 3; mean ± SD). Data for fibres without cross-linking are also included on the chart.

The number of cells adherent to the collagen fibre was observed to increase with decreasing cross-linking concentration (Fig. 5). This data was supported by the SEM images of cells adherent to collagen fibres after 1 week incubation. Cells on fibres from the 25 mM group (Fig. 5b) were generally rounded and few in number, whilst samples cross-linked with 0.25 mM EDC concentration showed more cells, with flattened morphology and cellular processes clearly evident. This was supported by a significant increase in cell proliferation with reduction in cross-linking concentration (Fig. 6). When the amount of cell DNA extracted from human tenocytes seeded on the various cross-linked scaffolds was considered, significance was observed (*P* < 0.05) between the 25 and 0 mM after 1 week, between 25 and 2.5 mM and 0 mM after 2 weeks and between all cross-linking concentrations, with the exception of 0.25 and 0 mM after 3 weeks.

## Discussion

During tissue engineering for tendon repair and regeneration, any construct must promote cell attachment and proliferation to allow the rapid synthesis of a new tissue matrix, before degrading over time. The degree of degradability must be carefully balanced to allow strength where necessary, yet prevent poor repair as a result of prolonged implant presence. These at time contradictory requirements necessitate careful optimization of the degree of cross-linking of collagen structures. With the cross-linking of collagen-based materials using carboxylic acid group activation methods such as EDC gaining considerable interest, it was felt that a study into the effect of concentration of this agent in combination with NHS on mechanics, stability and cellular response was required. This was particularly true for the specific application of collagen fibres in tendon repair.

Whilst the data in this article does not support any significant effect of cross-linking concentration on fibre mechanics, the resistance to enzymatic degradation and cellular response were heavily influenced. The data showed a significant increase in cell attachment to be associated with reduction in cross-linking concentration. SEM imaging additionally demonstrated that fewer, more rounded cells were observed on fibres with the 25 mM EDC cross-linking chemistry, compared with the 0.25 mM material where the cells exhibited a flattened more phenotypical appearance. This was supported further by an increase in the amount of DNA extracted.

There is a body of work that has investigated the effect of EDC/NHS cross-linking concentration on the mechanical characteristics of collagen structures. Whilst Powell *et al.* [52] found the ultimate tensile strength (UTS) of collagen-GAG scaffolds to scale positively up to 10 mM EDC, they then found a significant decrease between 10 and 50 mM EDC. Hafemann *et al.* investigated the EDC/NHS cross-linking of collagen/elastin membranes and found cross-linking of the membranes to have no relevant effect on mechanical features. Further, Olde Damink *et al.* [44] found a higher cross-linking concentration to result in a small but significant decrease in tensile strength. It has been hypothesized that at high cross-linking concentrations, the diffusion of the cross-linker into the collagen is slowed by the rapid initial cross-linking of the surface, limiting the efficacy of the cross-linking agent [52]. However, at least from one study it appears that an increase in molar ratio of EDC to NHS results in continued increase in degree of cross-linking, at least within the concentration range studied [44]. Although no significance was observed in any of the mechanical properties for the three cross-linking concentrations considered in this current work, the fibres appear to become stiffer (higher modulus and lower strain to failure) with increasing cross-linking concentration. It is possible that this supports the hypothesis of Olde Damink *et al.* [44], that the decrease in ultimate tensile strength with increased cross-linking was a result of local stress concentrations due to early failure of brittle collagen fibres formed after extensive cross-linking [44]. The current work, whilst suggesting that cross-linking concentration does not have a significant effect on mechanics, does not attempt to contradict the previous finding that fibres entirely without cross-linking observed a significantly reduced ultimate tensile strength compared with those with cross-linking [41].

When placed into bacterial collagenase, the non-cross-linked material had entirely degraded after 6 h, whereas the 2.5 mM and 0.25 mM material lasted 48 h and the material with the 25 mM cross-linking concentration remained even after 21 days. Degradation times with collagenase are not representative of degradation times after implantation, but can certainly act as an indicator of relative degradation rates between cross-linking concentrations post implantation. In a recent study by Enea *et al.* [24], collagen fibre constructs with a cross-linking concentration similar to the 25 mM EDC level were implanted into the central third of the patellar tendon in an ovine model. Implanted fibres exhibited very little degradation even after 6 months and this was felt to largely explain the relatively limited tissue regeneration observed. The current study suggests that use of 1/10 or even 1/100 of this cross-linking concentration may provide more favourable degradation and thus more significant tissue regeneration.

Consistently, degradation studies have shown even low degrees of EDC/NHS cross-linking to have a significant effect on the rate of enzymatic degradation compared with material without cross-linking [49-52]. In one study investigating the susceptibility of dermal sheep collagen cross-linked with EDC/NHS towards enzymatic degradation, non-cross-linked collagen had a degradation rate of 18.1% per hour, whilst no weight loss was observed in the cross-linked fibre over a 24 h period [50]. In a separate study, collagen matrices with just 2 mg/ml cross-linking revealed a partial degradation after 6 days of collagenase treatment whilst native collagen was entirely degraded after 2 h [51].

Contrary to the use of bifunctional agents such as glutaraldehydes, carbodiimide based routes such as the combination of EDC/NHS, do not result in incorporation of the agent into the material [44]. EDC itself is water soluble and the only by-product of the reaction, urea, is also soluble and non-toxic and as such an absence of cytotoxicity is generally assumed. *In vivo* studies with carbodiimide cross-linked collagen have generally yielded positive results. For example, van Wachem *et al.* [53] demonstrated that material cross-linked using this route and implanted subcutaneously into the rat, very rarely induced an increased infiltration of neutrophils or macrophages as compared with normal wound healing and that the degradation rate was such that an optimal rat collagen matrix was formed. Goldstein *et al.* [19] observed that 10 weeks after implantation in a rabbit Achilles tendon defect, the carbodiimide cross-linked implant had been resorbed and replaced by normal looking neotendon. Whilst there are studies that also suggest an absence of cytotoxicity *in vitro* [49, 54, 55], others have shown EDC and NHS cross-linking agents to have a negative effect on cellular attachment and proliferation. For example Hanthamrongwit *et al.* [56] observed EDC to be cytotoxic when scaffolds were not washed thoroughly, although this was with higher EDC concentrations than the maximum considered here. Another study found that the highest concentrations of 10 or 50 mM EDC produced cytotoxicity in a co-culture of human fibroblasts and keratinocytes with reduced viability and poor cellular organization. At low concentrations, however, cellular response was comparable to the non-cross-linked material [52].

Whilst a description of cross-linking quantities in terms of solution concentration appears to be the literature standard [22-24, 51-53, 56], it is not rigorous, as both solution volume and quantity of collagen affect the ratio of cross-linker to collagen. Perhaps a more useful approach is that of Olde Damink *et al.* [44] where the ratio of number of moles of cross-linking agent to moles of assumed carboxylic acid group on the collagen is given. In their work on dermal sheep collagen, they assume that each α-chain (1000 amino acids with a molecular weight of 100 000) contains 120 carboxylic acid residues [44]. Therefore in this current work, where 3 litres of cross-linking solution are used per 10 fibre bundles (with mass of collagen in each fibre bundle equal to 90 mg) it is possible to calculate the ratio of molecules of EDC and NHS to the number of carboxylic acid groups. These values are provided in Table 1. A ratio of 70:1 for EDC: carboxylic acid groups in the case of the standard cross-linking concentration is a significant excess of the cross-linking agents and may well explain the cytotoxicity of this material. More importantly perhaps, it also appears a significant waste of the costly cross-linking agents. Further *in vivo* tests looking at the effect of cross-linking concentrations are required in order to correlate collagenase resistance with *in situ* degradation and consequent healthy *in situ* neotendon formation.

**Table 1:** Ratio of molecules of cross-linking agents to collagen carboxylic acid groups

Cross-linking condition	Solution concentration (EDC, NHS)	Ratio of molecules EDC to number of carboxylic acid groups	Ratio of molecules NHS to number of carboxylic acid groups
25 mM	25 mM, 12.5 mM	70:1	28:1
2.5 mM	2.5 mM, 1.25 mM	7:1	2.8:1
0.25 mM	0.25 mM, 0.125 mM	0.7:1	0.28:1

Whilst this study has not identified the ideal cross-linking concentration for collagen fibres to be used in tendon augmentation, it does suggest that contrary to previous suggestions, a higher level of cross-linking may not produce a stronger fibre and that by using lower cross-linking concentrations a suitable balance of mechanics, degradability and biological response may be achieved. It is quite possible that even when cross-linking is optimized, the association between scaffolds, cell therapy and growth factors may well prove the key to successful tendon repair and regeneration [57].

## Conclusion

This work demonstrates that whilst the concentration of EDC and NHS cross-linking agents had no significant effect on fibre mechanics, a higher concentration was associated with higher collagenase resistance but also provided a less attractive surface for cell adherence and proliferation. Previous literature has suggested that fibre constructs highly cross-linked with EDC/NHS degraded too slowly for optimal tissue regeneration but suggested lesser cross-linking would result in a reduction of mechanics [27]. It appears however that with a lower cross-linking concentration, a more biocompatible material could be produced, without reduction in mechanics and with a potentially more optimal degradability.

## Supplementary Data

Additional data related to this publication is available at https://www.repository.cam.ac.uk/handle/1810/247492
